# PlatformTM, a standards-based data custodianship platform for translational medicine research

**DOI:** 10.1038/s41597-019-0156-9

**Published:** 2019-08-13

**Authors:** Ibrahim Emam, Vahid Elyasigomari, Alex Matthews, Stelios Pavlidis, Philippe Rocca-Serra, Florian Guitton, Denny Verbeeck, Lucinda Grainger, Erica Borgogni, Giuseppe Del Giudice, Mansoor Saqi, Paul Houston, Yike Guo

**Affiliations:** 1 Data Science Institute, Imperial College London, London, UK; 2 Clinical Research Centre, University of Surrey, Guildford, UK; 3 Oxford e-Research Centre, University of Oxford, Oxford, UK; 4 Janssen Pharmaceutica NV, Beerse, Belgium; 5 GSK Vaccines Srl, Siena, Italy; 6 CDISC, Clinical Data Interchange Standards Consortium and CDISC EU Foundation, London, UK

**Keywords:** Translational research, Standards, Data integration, Research management

## Abstract

Biomedical informatics has traditionally adopted a linear view of the informatics process (collect, store and analyse) in translational medicine (TM) studies; focusing primarily on the challenges in data integration and analysis. However, a data management challenge presents itself with the new lifecycle view of data emphasized by the recent calls for data re-use, long term data preservation, and data sharing. There is currently a lack of dedicated infrastructure focused on the ‘manageability’ of the data lifecycle in TM research between data collection and analysis. Current community efforts towards establishing a culture for open science prompt the creation of a data custodianship environment for management of TM data assets to support data reuse and reproducibility of research results. Here we present the development of a lifecycle-based methodology to create a metadata management framework based on community driven standards for standardisation, consolidation and integration of TM research data. Based on this framework, we also present the development of a new platform (PlatformTM) focused on managing the lifecycle for translational research data assets.

## Introduction

Translational research (TR) is often described as a data intensive discipline. An intrinsic complexity in the translational approach is brought by the granularity, scale and diversity of data collected and observed during a study. Collected data include phenotype data, such as demographics, diagnosis, lab tests, clinical events, medications as well as sample (specimen) data are collected during clinical trials and hospital encounters. Moreover, high dimensional datasets are generated from molecular profiling including genomics, transcriptomics, proteomics, and metabolomics techniques, which are becoming routine.

Challenges in integration and analysis of such diverse and voluminous data led to the emergence of Translational Bioinformatics (TBI)^1,2^. TBI tool and infrastructure developments have consequently adopted an analysis-driven informatics approach. Recent reviews of non-commercial TBI solutions^3,4^ demonstrate their success in enabling TR studies to conduct integrative analysis, generation and validation of complex hypotheses, data exploration and cohort discovery^5^. Essentially, these platforms focus on supporting the analytical requirements of a research project ensuring its scientific goals are met during its short-term life span. Other platforms such as dbGap^6^ and ImmPort^7^ offer a data archive to preserve data after the termination of a project, but they do not play a role during its active phase.

**Fig. 1:**
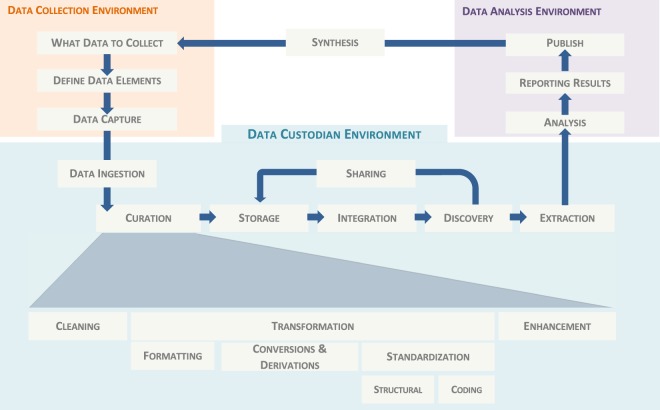
Research data life cycle highlighting in detail the different stages of the data pipeline between data collection and data analysis, the scope for the proposed data custodianship environment.

Data integration and analysis, viewed in light of the data lifecycle^8^, are only part of a more elaborate process to collect, curate, store, integrate, find, retrieve, analyse, and share data (Fig. 1). Together, these stages form a non-linear data pipeline, which often involves a lot of communication and feedback between different user roles, including curators, data managers, clinicians and bioinformaticians. Managing research data assets throughout this data pipeline presents another challenge, namely a data management challenge, towards improving the efficiency of the research process, and achieving the vision for the reuse and long term preservation of data.

Furthermore, there is an increasingly widespread recognition that data could potentially be reused and repurposed to support new areas of research. Academic initiatives, such as the Denton Declaration for open data recognises the role of open data and open access to research data as being critical for advancing science, scholarship and society^9^. With this recognition comes increasing pressure for researchers to do more with their data to ensure its availability and utility for purposes outside of the context in which they were originally generated. More recently, four foundational principles: Findability, Accessibility, Interoperability, and Re-usability (FAIR)^10^ have become the guiding means towards achieving successful reuse of scholarly data.

There is currently a gap in the informatics literature focusing on the ‘active management’ of data assets during the lifetime of a research project. In translational research studies ﻿data tend to be produced and administered “in the wild,” meaning that researchers typically devote very little consideration to how the data could be used beyond its initial purpose. Leaving data management planning as an afterthought severely reduces the long term value of research data assets and limits the potential for reuse. ﻿In data governance, data custodianship pertains to applied methodologies and infrastructure to manage the usage and preserve the value of data. We believe that establishing a data custodianship environment is essential to promote the importance of managing data as assets independent of specific analytical needs, nevertheless improving the analysis and reproducibility of research results. It will also ensure that data conforms to the FAIR data principles, which are established and reinstated by decisions and actions taken at each stage of the data lifecycle governed within this environment. The FAIR data principles are guidelines that define the criteria for achieving shareable and reusable data. However, this still leaves the details of how to actually achieve this in practice.

Various stages of the data life cycle in TM research, such as discovering, reusing, sharing, and analysing data are entirely dependent on the use of metadata and data standards. Several recent reviews suggest that a major challenge in translational bioinformatics is the lack of adoption of such standards that is often due to barriers in understanding, navigating and using these standards^11–13^. This paper begins by describing a data lifecycle management approach to develop a standard-compliant metadata management framework for translational medicine research. This framework is designed to incorporate different types of metadata models, i.e. administrative, descriptive, structural, and provenance metadata within the context of translational research domain. Based on this framework, we then present the design, development, and application of PlatformTM: a standards-compliant data custodianship environment for all user roles involved in managing the data lifecycle between data collection and data analysis. Focusing on the ‘active management’ of data, the platform provides a set of core functionalities to handle metadata definition, file management and data loading, data storage, retrieval, visualization, export and data sharing. Underlying these set of features is an integrated solution that includes a data repository for consolidating and archiving primary datasets, a data warehouse for integrated and harmonized clinical and molecular observations, and user-based database for sharing analysis-ready datasets.

## Methods

### Characterization of translational research data lifecycle

From its inception to its use and completion, research data will likely undergo multiple transformations in its format, application, use, and perhaps even its purpose. Data lifecycle management focuses on the data itself as assets and acknowledges that managing data requires managing its lifecycle. We adopted a data lifecycle model to identify and characterize the transformations that data will undergo through processing as stages in a larger lifecycle ﻿from origination to usage. These stages are:

1. **Data elements**: a question-answer pair representing the formalization of data at the most granular level at the stage of data design and planning. Formalized by the international standard ISO/IEC 11179, a data element provides the meaning behind a measurement by associating its data *value* (e.g. 27) with a *concept* (e.g. Age). Functionally, a data element carries the definitional information about any given data value, and as such, data elements can be considered the atomic unit of information.
2. **Raw data files**: during acquisition stage of the data life cycle, data gets created by obtaining *data values* for the set of planned and designed *data elements* to form raw data usually in the form of data files. Raw data can exist in many different forms and structures, which are optimised for managing the acquisition process within the environment or system generating them.
3. **Primary datasets** are structurally and semantically *annotated datasets* according to a standard data model designed to organize and consolidate data into a common reusable form. As managed data assets they are optimized for data manipulation and data curation functions, preparedness for data integration and long term storage, data sharing and reuse.
4. **Integrated data**: A state of persistent data whereby content from all primary datasets is integrated into a common data model for purpose of querying and asking research questions at data.
5. **Analysis-ready datasets**: essentially represent the *usage* of integrated data when sliced and diced for a specific analysis. Analysis datasets are reformatted, hypothesis-focused datasets optimised to efficiently generate and report analysis results to support research reproducibility.

**Fig. 2:**
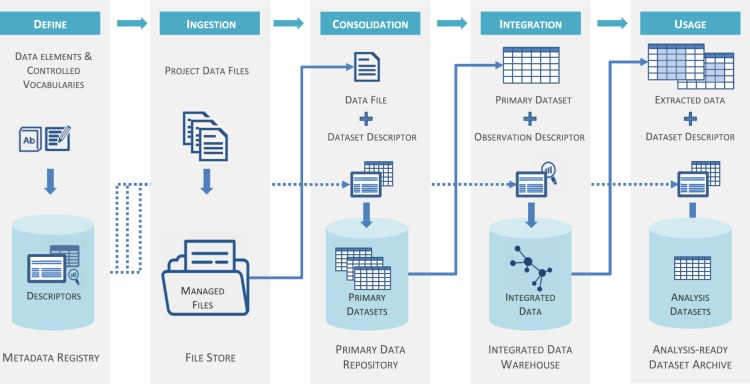
Our proposed data lineage management workflow. Each stage has its own data form, data service (top blue boxes) and data storage resource (bottom grey boxes). Decisions about what data to collect start with the formulation of research questions (modelled as data elements) and culminate in data collection to collect values for specific data elements to produce raw data files. Files are then semantically and structurally annotated by dataset descriptors and consolidated into primary datasets. Content from all datasets is then integrated according to a common observation model each observation semantically defined by an observation descriptor. Finally, user queried data is extracted and saved to analysis-ready annotated datasets.

Figure 2 illustrates the lineage of data through these different lifecycle stages and forms. This lays the foundation to our approach to identify and describe component data management services and resources that are necessary for data custodianship. We also use this approach to define the metadata necessary to manage data at each stage, which we developed into a metadata framework discussed below.

### Implementation of data and metadata standards

One of the key functions of a data custodianship environment is to implement an enforcement mechanism to use data and metadata standards bringing all relevant existing standards into a common framework. The eTRIKS standards starter pack^14^ for guidance on the adoption and use of data standards relevant to TR recommends the use of standards from the Clinical Data Interchange Standards Consortium (CDISC) (https://www.cdisc.org/standards) to describe data at different stages of the clinical research pipeline. Similarly, in the domain of molecular assays (‘omics), the ISA model^15^ is a recommended community driven standard for describing assays across different technologies. Translational research data management relies on bridging both worlds. The design of our framework (described below) is influenced by the Meta-Object-Facility (MOF) specification (https://www.omg.org/spec/MOF), the CDISC standards (SDTM, SDM-XML, PRM), the ISA-TAB specification^16^ and the Observation pattern developed by Fowler and Odell^17^.

### TREMF: The Translational Research Metadata Framework

**Fig. 3:**
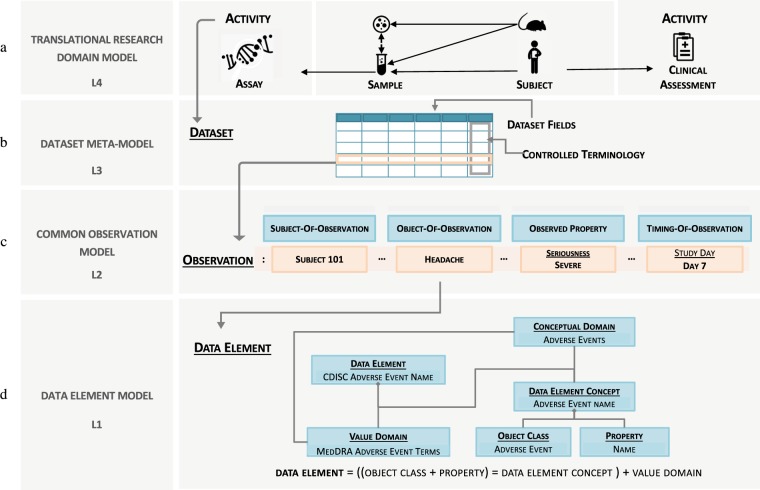
Translational Research Metadata Framework (TREMF). The domain model (L4) defines the common elements of a translational research project and the relations between them establishing context for exploring data and cross-study comparisons. Different activities (clinical or assays) within a project generate datasets that are modelled according to generic interoperable meta-model (L3). Dataset content (observational data) is modelled against the common observation model (L2): a vector of related data elements each defined according to the ISO/IEC 11179 data definition model part of the standard model for metadata registries (L1).

The centrepiece of the data management framework for data custodianship is the development of the Translational Research Metadata Framework (TREMF) (Fig. 3). It comprises tiered data models to (1) understand the framework’s data assets (2) model how data assets fit together and (3) define the context for the data management framework. Each tier is concerned with a different aspect of metadata management. Collectively, they form a comprehensive metadata management framework for the standardisation, consolidation, and integration of translational research data.

The top layer is the domain model describing the study, its main elements and the relationships between them. It establishes the context for data *consolidation*. The third layer is the ‘Dataset meta-model’: a multi-layered meta-model based on community standards describing data in the form of a ‘dataset’ to support standardisation and interoperability of data exchange. The second layer is the ‘common observation model’ describing data in the form of an ‘observation’ to support data *integration* for analytical queries. The bottom layer is a data definition layer that describes each data element to enable interpretation, communication and processing of data. In the following subsections, we discuss bottom up each layer in details.

#### Data element metadata model

Defining data is describing each data element in a way that leaves no ambiguity for humans or computers. This first layer of the model lays the foundation for defining all constructs of data for the purpose of interpretation and processing of data semantics. We adopted the ISO/IEC 11179 which defines a data element as an association of a concept (called a data element concept) with a value domain. For discrete data elements the value domain comprises the possible values that might be collected about the concept. For continuous data elements, the value domain is defined in terms of the type of data, a valid range and the unit of measure. Full specification is available at (http://metadata-standards.org/11179/).

#### Common observation model

One of the challenges with usage of data in handling *integrated* observational data for querying, visualization or simply browsing them is the need to get a higher level view of the data content with the ability to systematically drill down into the details thus allowing a manageable stepping down into the data. The main goal of the *common observation model* is to provide a model for structuring and semantically describing integrated data and related contextual data to enable researchers to navigate very large amounts of heterogeneously captured data with a consistent and systematic approach.

The key idea behind the common observation model is the underlying presumption that an ‘Observation’ cannot be modelled as a simple fact-attribute concept, rather it consists of a set of semantically related data elements that together provide the desired contextual information necessary for the interpretation of a phenomenon of interest. It also enables the separation of the metadata elements describing the observation from the values measured/observed which in many cases is necessary for security and access management.

Similar to a phrase or a sentence the common observation model deconstructs an instance of an observation into the following construct data elements:

- Object-of-observation: the feature being observed, whether in a clinical or molecular setup; e.g., weight, albumin, headache, TP53, CD40
- Subject-of-observation: the entity upon which the observation is being observed
- Observed property: qualitative or quantitative property of the observed feature being observed or measured; e.g. count, result of test, severity of headache, amount of dosage
- Temporal properties: timing attributes that are not longitudinal such as time of collection, duration, start of event, interval …etc.
- Time-series properties: properties that cause the repetition of the same observation over time, resulting in a longitudinal observation; e.g. visit, planned study day, time point.

#### Dataset meta-model

The third layer of the framework comprises the dataset meta-model, which describes the metadata used to describe primary datasets. As previously explained, primary datasets provide a standardized way to group and organize observations and other study data into structurally and semantically annotated datasets to facilitate three key data management functions: data curation, data consolidation and data exchange.

**Fig. 4:**
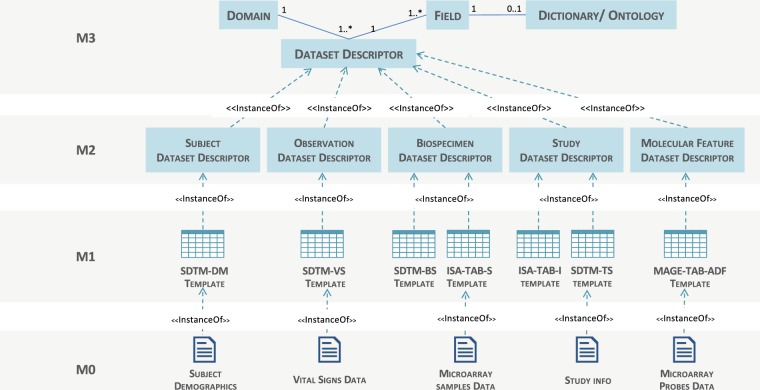
Dataset four-layer metamodel hierarchical architecture for dataset interoperability. Data at M0 level, such as subject demographics, are described by models at the M1 level, such as CDISC SDTM dataset format, which in turn are described by metamodels at the M2 interoperability level, such as subject dataset descriptor, which in turn conforms to a generic dataset descriptor model described in M3 layer.

Therefore it is essential to maintain extensibility for new dataset types and to achieve semantic interoperability between different formats for datasets of the same type. For this reason we adopted a layered metamodeling approach based on the classical four layer metamodeling architecture^18^ where elements in a given layer describe elements in the next layer down. The key benefit of this metamodeling approach is that metadata that define the structure and semantics of specific *types* of dataset (i.e. metamodel layers) is separate from metadata that enforces a concrete syntax or notation (i.e. data model layer). SDTM demographics dataset, SDTM medical history dataset, and ISA-TAB transcriptomic assay dataset are examples of a primary dataset models. Each dataset model conforms to a meta-model described by a dataset descriptor, which defines its domain, the structure of the dataset, the syntax and semantic of its fields, any enforced controlled vocabularies, and validation rules. The hierarchy of defining a dataset descriptor is illustrated in Fig. 4 and explained below:

1. The *meta-metamodel* layer (M3) describes the generic dataset descriptor. It encompasses the necessary metadata to describe *any* dataset. It defines metadata to describe the *structure* of dataset annotating each column/field of the dataset. The dataset descriptor also provides the means to manage the *content* of a dataset by associating each field with a dictionary of terms drawn from standard terminologies or ontologies. This has a role in the harmonization and standardisation of data between different datasets.
2. Informed by the layer above, this *metamodel* layer (M2) is comprised of the descriptions that define the structure and semantics of common *types* of datasets, i.e. meta-descriptors. This means that all dataset models implementing a particular meta-descriptor would be considered semantically equivalent and interoperable, even though they might be syntactically represented differently. This layer essentially maintains the semantic interoperability for various datasets. Meta-descriptors in this metamodel are based on a conceptualization of the core elements that are expected to be in any translational research project rather than based on experimental features that might be methodology or technology specific. These core elements are: the study, the subject, the observation, and for molecular assays: the bio-sample and the molecular observable features. For each of these elements a dataset meta-descriptor is defined as shown in Fig. 4.
3. Informed by the layer above, this *data model* layer (M1) is comprised of the metadata that describes data in the information layer. To ensure that the data exchange process is maximally interoperable with other external databases as well as to encourage data curators to adopt and implement data standards as part of their data management procedures, the data model prescribed in this layer is an aggregation of clinical and mechanistic data models from sources including Clinical Data Interchange Standards Consortium (CDISC; www.cdisc.org/) and Investigation Study Assay (ISA-TAB) specification (https://isa-tools.org/format/specification.html) to support clinical and ‘omics data exchange respectively.
4. The information layer (M0) comprises the actual data we wish to describe (e.g. demographics, medical history or biospecimen data).

#### Translational research domain model

This top layer model describes the core elements that are common to the domain of a translational research study and the relationships between them. Based on a generalization of the CDISC Study Design Model (SDM) and the ISA data model, we created a hybrid model that integrates standards for reporting data and meta-data elements for clinical assessments and biomarker/omics assays (Fig. 3a). The model comprises classes such as, subjects, activities (clinical assessments or molecular assays), bio-samples, study design elements and the relationships between them. Most importantly for any translational study, it establishes relationship between data captured from human subjects, to their *in vitro* specimen experiments and in some cases animal model *in vivo* experiments. Another notable relationship is between class ‘activity’ and class ‘dataset’. Under this model, all data generated from an activity is modelled as a ‘dataset’, which is defined by the third layer of the TREMF (Fig. 3b). Adhering to the TR domain model maintains a consistent representation of TR studies and enables cross-study data integration by establishing the study context for *consolidating* and relating the observed data to it.

### Data consolidation

Data consolidation is the data management process associated with managing data during its life cycle stage as primary datasets. Perhaps the most critical gap in the data lifecycle for translational research projects is between the stages where data are actively being collected or acquired from different data collection environments to where the data transition into being curated, integrated and eventually analysed. A key component of data custodianship is establishing a ‘primary data repository’. It provides a study-centric data hub for consolidating research *primary datasets* during the active stages of the data life cycle, ensuring the data’s integrity, consistency and completeness before any analysis takes place. It also offers researchers a trusted ‘single source of truth’ for sharing and preserving data for reuse once in an inactive stage. Unfortunately, data consolidation is omitted in most translational research projects, where data marts, or star-schema data warehouses are populated directly after initial data cleansing. These databases provide a denormalized projection of data which is optimized for querying and analysis of integrated data, nevertheless not suited for preserving the integrity of data for long term use or re-usability outside the project’s specific needs.

Our data consolidation process depends on 1) a standard exchange model ﻿into which data from source systems is extracted as a prelude to consolidation and 2) a ﻿consolidated master representation model to act as the core repository. ﻿The exchange model we implemented is based on the dataset metamodel (TREMF layer 3). As previously described, it is a generalized meta-model that can have different standard implementations. To this end we developed a set of standard compliant data exchange templates covering all CDISC SDTM domains (findings, events and interventions) and preloaded them into the database. Similarly, for assays, we preloaded standard templates for sample and feature metadata based on ISA model and assay measured data catering for different types of assay technologies; e.g. microarray gene expression, flow cytometry, proteomic and immuno assays. Each dataset ingested into the data repository is associated with a predefined standard dataset descriptor which will include metadata necessary to describe each dataset’s structure, syntax, semantics and content. Datasets are consolidated and persisted according to the TR domain model (TREMF layer 4) providing a consistent study-centric relational model for relating datasets to the research project design and context thus establishing a primary consolidated resource for a project’s research data.

### Data integration

Data integration refers to the process that extracts data content from the primary datasets and transform it into queryable, explorable and navigable data irrespective of the different dataset standard templates used for data ingestion. As a result, the researcher’s experience for data query and visualization is not influenced by the data manager’s choices of standard dataset formats.

Following the data consolidation process, data from each dataset is extracted and mapped to the common observation model (TREMF layer 2). Data at this stage becomes integrated across different domains (demographics, diagnosis, laboratory tests, medications, etc.); across studies (clinical trials); and if molecular data is measured, across multiple omics platforms linking subject omics data to phenotype data. The resulting integrated data is loaded into a data warehouse whose schema is based on common observation model. No separate ETL process is required to move data between the data repository and the data warehouse, which mitigates the inefficiencies often associated with moving data between different implementations. This is due to the meta-modelling approach discussed earlier, which adds the necessary semantics to the primary dataset to extract its content into the common observation model during the data integration process.

## Results

As stated in the introduction, we adopted a lifecycle management approach to develop a comprehensive and effective metadata framework to manage the translational research data through its different lifecycle stages. We used this framework to develop PlatformTM: a proof-of-concept implementation of a data custodianship environment for managing the data lifecycle between data collection and data analysis. In the following sections, we present in details the platform’s different modules and features (Supplementary Figs 1 & 2). PlatformTM system architecture and implementation are described in Supplementary File 1.

### Metadata governance

The metadata governance module offers data managers a set of features via a simple and intuitive dashboard to define and manage elements of metadata. This includes defining the research project against TREMF layer 4 (Fig. 3a) and the acquired data against TREMF layer 3 (Fig. 3b).

Setting up a project from the metadata governance dashboard is the entry point into the system. A project can be a single study, a multi-study (planned related studies), or a meta-study (unrelated studies). The ‘studies panel’ enables managers to enter information about each study within a project such as study design, eligibility criteria, objectives and other study metadata elements via a web form compliant with the CDISC Protocol Representation Model (PRM) and CDISC Study Design Model (SDM). The ‘activity panel’ allows the data manager to create and manage the project’s planned clinical activities, molecular assays and their associated dataset descriptors, while the ‘Members and Users’ panel is used to manage user roles and their data access rights.

**Fig. 5:**
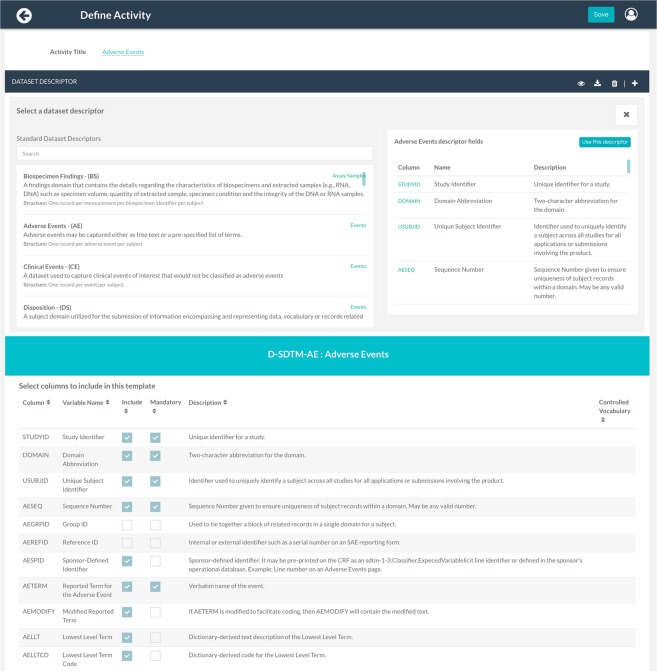
Metadata Governance module. For each project activity, a standard-based predefined dataset descriptor is created to define metadata for a primary dataset. First users can browse and search through all preloaded templates. Once a template is selected, user can then customize the structure of the dataset to fit their data.

‘Manage activity’ page (Fig. 5) allows a data manager to create and edit dataset descriptors (dataset metadata) for each planned activity based on the preloaded standard templates. Features include excluding/including fields, setting mandatory fields, specifying controlled vocabularies for a field’s permissible values, as well as adding new fields.

### File management and data loading

**Fig. 6:**
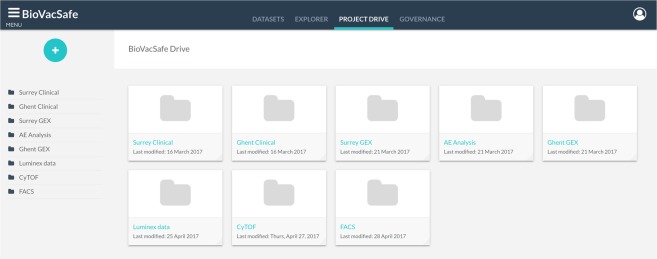
Project Drive. This module organizes all uploaded project files and manages the loading process into the platform’s databases.

This module provides users with the right credentials to upload and manage the project’s data files. Similar to online storage drives, an automated audit trail functionality tracks the creation of new data files, changes to existing ones, loading status, the user initiating the action, and data and time of the action (Fig. 6). Besides organizing the project’s files, the project drive is the entry point for loading data into the platform’s databases described above. This is the process whereby data files become associated with standard dataset descriptors and hence become primary datasets. To load a file, the user launches the loading wizard which takes them through a series of steps to associate the file with one of the previously defined dataset descriptors. The descriptor is used by the loading process as a reference for parsing and validating the file contents accordingly. Once validated, the loading process persists the file as an annotated primary dataset into the data repository, followed by the consolidation and integration processes discussed earlier to extract the dataset content and load it into the integrated data warehouse as illustrated in Fig. 2. The loading wizard natively supports CDISC SDTM formatted files.

### Data retrieval & exploration

#### Datasets access

**Fig. 7:**
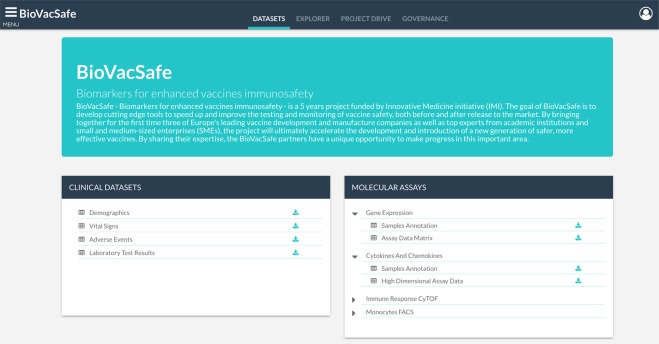
Datasets module. Browse and download project’s consolidated datasets.

This module offers a graphical user interface that enables users to browse the set of projects stored in the *data repository*, enabling the retrieval of structurally and semantically annotated *primary* datasets for sharing and reuse. For each stored project, a project summary page provides metadata about the project and links to the associated datasets available to download (Fig. 7).

#### Data explorer & query module

**Fig. 8:**
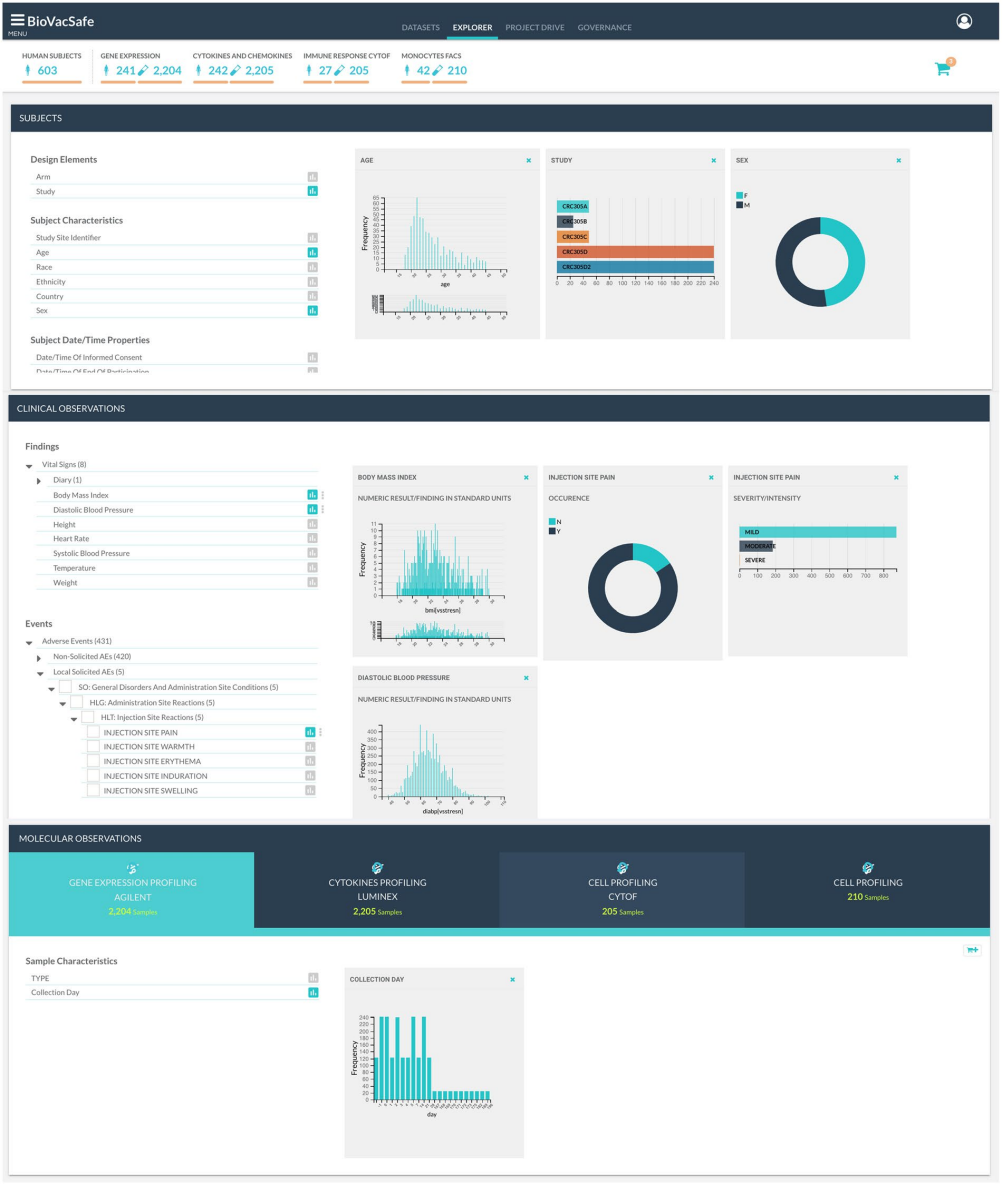
Data explorer and query interface. (**a**) Subject Panel, (**b**) Clinical data panel, (**c**) Molecular observations panel. Each panel lists observation features (metadata) on the left and data plots for each clicked observation on the right. Filtering data through the plots, subjects and samples satisfying the filters are automatically updated on top. Clicking on the cart icon in the top right corner lists all observations selected with an option to checkout and generate analysis-ready dataset for the queried data.

The data explorer is a visual browser and query interface supporting ‘slice and dice’ exploration and retrieval of integrated data stored in a *data warehouse*. It uses on-demand synchronized charts to provide a hypothesis-free, interactive, and easy-to-use graphical interface to explore integrated subject and sample related observations. This is particularly useful during the initial phases of research when no clear hypotheses are immediately available. The layout design is based on an domain-aware visual layout organizing data across three panels (Fig. 8). The first panel hosts subject and study data elements, such as subject demographics, study arms, visits …etc. The second panel is for exploring the integrated clinical observation features organized by the CDISC general observation class-domain hierarchy, while the third panel is for molecular observations organized by assay type. The three panels offer a faceted browsing component for the related data elements and an interactive dashboard showing a chart for each selected data element or observation feature. Metadata of the observations that make up the content of the faceted panels is retrieved from a different database than the one used to retrieve data for charting the data values essentially giving the option to restrict access rights to data values if required.

Besides offering a visual dashboard for browsing and localizing interesting features in the data, the ‘data explorer’ acts as a visual query builder to support hypothesis generation through interactive data selection and filtering directly off the charts. All charts across the three panels are synchronized. Adding a chart for an observation or a data element essentially adds it to the query. Applying filtering on any chart automatically cascades the effect of the filter to all other visualized charts effectively affecting the number of subjects and samples currently satisfied by the query. For example, filtering for a particular range of ‘diastolic blood pressure’ followed by applying a filter on ‘study arm’ and ‘visit’ will automatically be propagated to the related omic assays, reducing them to only the matching filtered subjects and vice versa. In addition to the plotted charts, a count panel displayed on top is dynamically updated to show the number of subjects, samples and assays satisfying the selected and filtered data.

A ‘data cart’ feature in the data explorer module allows the user to save their queries to retrieve later or to ‘checkout’ whereby the query results are exported into analysis datasets. At checkout, the server prepares the data exports according to the data query giving the user the option to add their own descriptions and tags before they are ready to download or save to their analysis datasets library. An analysis dataset stores the query that the user specified to extract the required data rather than the actual resulting data. This allows export files to be automatically generated every time there are changes to the primary data source including the contents of any derived fields. The analysis dataset also holds general metadata information about its contents, as well as field-level metadata describing the columns in each of its data files. Sharing permissions enable users to share or publish their datasets promoting open data ethics and reproducibility of research results. Analysis datasets are stored in a separate storage collection that is user-focused and not project based. They are accessible via the API using their unique URL to download associated export files. The analysis datasets library page provides a user workspace to manage their own created datasets, shared datasets from other users and published datasets made public by other users.

### Case-studies

The work presented here was developed at Imperial College London Data Science Institute (ICL-DSI) as part of its collaborations with the Innovative Medicine Initiative (IMI) (https://www.imi.europa.eu/) funded projects: eTRIKS and BioVacSafe. eTRIKS is a service project aimed at supporting data management and analytical needs of other IMI projects through the delivery of an open sustainable translational research informatics and knowledge management platform. PlatformTM was developed to support data management services for the eTRIKS platform. Throughout the eTRIKS project, we had the opportunity to gather requirements and learn about real problems drawn from many eTRIKS supported projects such as U-BIOPRED^19^, OncoTrack^20^, PreDiCT-TB^21^ to cite a few. Here we present our proof-of-concept implementation drawn from one of the eTRIKS supported projects. We also present the first production implementation of the platform developed for BioVacSafe (Biomarkers for Enhanced Vaccine Safety)^22^: an IMI funded project that investigates vaccine reactogenicity to enhance immunosafety of novel vaccines.

#### The ERS case study

To demonstrate the applicability of the developed framework and the usability of our platform in supporting cross-study research and re-use of data, we conducted a pilot study with the European Respiratory Society (ERS) to compare various subpopulations of asthma and COPD patients from two independent studies: ‘U-BIOPRED’ (Unbiased BIOmarkers in PREDiction of respiratory disease outcomes) and ‘EvA’ (Emphysema versus Airway disease) respectively. Using the metadata module, a meta-study project was created for the pilot, defining two studies with different subject cohorts, four clinical activities: laboratory tests, vital signs, spirometry and reversibility tests, and a gene expression assay. For each activity, a dataset descriptor was pre-defined based on one of the preloaded CDISC SDTM standard templates to guarantee that overlapping clinical variables are uniquely represented across the two studies. Data files selected for the pilot were then uploaded to the dedicated project drive space and each loaded into the data repository and data-warehouse simultaneously via the loading wizard. Once loaded, data from a total of 1,294 subjects and 39 unique and harmonized clinical variables were readily integrated in the observation data-warehouse. Using the data explorer, lead investigators were able to explore the integrated data without requiring any programming background, come up with hypotheses using visually coordinated plots of the clinical features of interest, and determine instantly whether there are sufficient samples available to conduct a certain analysis, and finally save and extract the desired subsets ready to be analyzed. One of these hypotheses was to test if asthma and COPD sufferers with abnormally high eosinophil cell count and airflow obstruction share similar gene expression profiles. This proof-of-concept demonstrated the feasibility of reusing data for secondary research gathered from two independent consortia by utilizing our platform and its underlying metadata framework.

#### BioVacSafe Data Management System

Following the ERS case study, we continued developing PlatformTM as part of delivering a data management system for the BioVacSafe project. BioVacSafe is a multi-study and multi-site project that generated clinical, pre-clinical and ‘omics data for assessment of vaccine responses with an emphasis on immunosafety and immunogenicity^22^. Data were collected and stored from 2 different sites, running 5 clinical trials investigating 7 different cohorts with overlapping clinical and molecular observations. Clinical data^23^ included subject demographics, laboratory tests (haematology, urinalysis, chemistry), vital signs and MedDRA coded adverse events (solicited and non-solicited). Data from molecular assays included: microarray gene expression profiling^24^, cytokine/chemokine profiling^25^ and Immunophenotyping of Monocytes using FACS flow cytometry^26^. The platform and its underlying metadata framework provided a systematic and standard compliant approach to streamline the process of data consolidation and integration across the consortia’s work streams. Once loaded, the explorer module also offered researchers a systematic hypothesis-free method to navigate through the whole range of data, and to export different research focused analysis datasets. For instance, a common exploratory use-case was to select subjects based on some combined clinical observations specifying a potential reactogenicity profile, and export their corresponding assay data to run differential analysis to look for correlated molecular signatures.

## Discussion

This paper argues that translational informatics efforts should shift from being analysis-driven to become data lifecycle-driven. This shift acknowledges research data objects as assets of value that go through different lifecycle stages and take different forms throughout the research process. This approach improves the manageability of these assets and subsequently should improve the efficiency of the research process as a whole. It also facilitates sharing and re-use of data thereby more effectively exploiting the often costly investment that went into the data generation. Without a process and a framework for active management of TR data assets, well-intentioned goals and policies for improved sharing of research data will not succeed. We believe that establishing a data custodianship environment is essential to promote the importance of managing data as assets independent of specific analytical needs, nevertheless improving the analysis and reproducibility of research results.

Managing the data lifecycle between data collection and data analysis is a complex one involving different user roles, different tasks and different inputs and outputs. To solve this problem we sought in this paper to create a lifecycle methodology to develop a new multi-layered metadata framework to organize and describe the process of data management for translational medicine research studies. Based on this framework, we created PlatformTM as a first translational informatics platform focused on TM data management offering a common platform for the different tasks involved in the data pipeline between data collection and data analysis.

In its strategic research agenda, the IMI has highlighted the need for building an integrated biomedical data platform and interactive scientific exploration tools for their European public-private partnerships^27^. tranSMART^28^ is an open source knowledge TM management platform that enables scientists to develop and refine research hypotheses. tranSMART was recognized as the candidate solution to establish this IMI-wide platform. However, tranSMART was designed to support cohort-based analyses by offering a data warehouse model for integration of data based on the i2b2 Entity-Attribute-Value (EAV) star schema^29^. In light of the data lifecycle view, tranSMART is well placed for later analytical stages but it cannot provide the necessary metadata or underlying infra-structure to support the whole data pipeline as described earlier. However, a data pipeline using PlatformTM API could be easily established to push data into a tranSMART based analytical environment. The IMI envisions a platform that will allow data to be searched, queried, extracted, integrated and shared in a scientifically and semantically consistent manner. We believe that PlatformTM offers a solution that is in line with the vision that IMI has projected for the future of data and knowledge management in translational medicine research.

In the next few years, we foresee research data management to become an essential component of the research process. We envisage a network of data custodianship environments will be established to promote data reuse and the sustainability of data beyond single use. In its current implementation, the platform takes a few steps towards achieving this goal by giving individual projects their own local environment to manage and share their data. Future work will focus on extending the platform to support a hub and spoke implementation. We have already started adding a data curation module, which will enable data curators to transform and tabulate non-standard data files against the standard templates. This module utilizes a dedicated metadata registry (MDR) that we have built in compliance with the ISO 11179 standard for metadata registries to provide the necessary management for data elements. We have also set up a controlled vocabulary service based on the Ontology Look-up Service (OLS)^30^ to facilitate and enhance the data curation tasks. Future work will also concentrate on enhancing the performance of the platform, building data import pipelines from popular data capturing systems as well as data publishing pipelines to link to data journals.

## Supplementary Information

Supplementary Information.

## Data Availability

The datasets used for the use-cases are available in Harvard Dataverse with the identifier(s) 10.7910/DVN/QPHMKX, 10.7910/DVN/SCFQ1F, 10.7910/DVN/CKSLGB and 10.7910/DVN/34EMZ6.

## Code Availability

Source code and Docker distribution is freely available for download at https://github.com/dsi-icl/PlatformTM, implemented in C#, Javascript, MariaDB, MongoDB and supported on all platforms. Public demo instance available at: platformtm.cloud/app.
