# Supplementary material for: PlatformTM, a standards-based data custodianship platform for translational medicine research
Source: Sci Data. 2019 Aug 13;6:149. doi: 10.1038/s41597-019-0156-9 (PMC6692384; doi:10.1038/s41597-019-0156-9)
Supplement: Supplementary file 1 — Supplementary Information. [file 41597_2019_156_MOESM1_ESM.docx]

Table of Contents

[System Architecture & Implementation 2](#_Toc5275356)

System Architecture & Implementation

PlatformTM is a web-based application including a client-side application based on AngularJS framework and a server-side application based on the new cross-platform ASP.NET Core framework (Supplementary Figure 1). The server-side exposes RESTful API enabling communication with the front-end client application and other third party tools, while the client-side application provides an interactive user-friendly interface for data loading, querying, visualization and export. To maintain a balance between the flexibility of data acquisition and consistency of data and meta-data representation, we implemented a two-way storage solution. A relational database based on MariaDB is used to store the domain model described by TREMF layer 4 and the observation-based data warehouse based on TREMF layer 2, while a NoSQL database using MongoDB is used to archive loaded annotated datasets as submitted according to TREMF layer 3.

For data exploration and visualization, the application uses Crossfilter.js (http://crossfilter.github.io/crossfilter), a JavaScript library for exploring large multivariate datasets in the browser and DC.js (https://dc-js.github.io/dc.js/), a JavaScript dimensional charting library with native Crossfilter support allowing highly efficient exploration on large multi-dimensional dataset. Charts rendered using dc.js are naturally data driven and reactive therefore providing instant feedback on user’s interaction.

Supplementary Figure 1 PlatformTM software architecture

The software architecture of PlatformTM is based on a loosely coupled backend service application and a frontend client application communicating via REST-based web API (Application Programming Interface). Supporting the extendibility of the platform, we envisage this design to encourage further development of new applications as well as third-party integrations with other platforms. Incorporating PlatformTM data into any external app requires a HTTP library and a JSON parser. HTTPS is supported for secure client access. PlatformTM API is organized into three distinct API collections: The ‘Datasets API’ provides read-only accessibility to the primary data repository allowing third party applications to find and access meta-data rich translational medicine research datasets. The ‘Observation query API’ exposes endpoints to query the data warehouse: selecting, filtering and combining data from all integrated domains of data within a project. The ‘Apps API’ is a client dependent API serving the frontend PlatformTM client web application (Supplementary Figure 2).

Supplementary Figure 2 PlatformTM application modules
